# Interference with histidyl-tRNA synthetase by a CRISPR spacer sequence as a factor in the evolution of *Pelobacter carbinolicus*

**DOI:** 10.1186/1471-2148-10-230

**Published:** 2010-07-28

**Authors:** Muktak Aklujkar, Derek R Lovley

**Affiliations:** 1 University of Massachusetts Amherst, Amherst, MA, 01003, USA

## Abstract

**Background:**

*Pelobacter carbinolicus*, a bacterium of the family *Geobacteraceae*, cannot reduce Fe(III) directly or produce electricity like its relatives. How *P. carbinolicus *evolved is an intriguing problem. The genome of *P. carbinolicus *contains clustered regularly interspaced short palindromic repeats (CRISPR) separated by unique spacer sequences, which recent studies have shown to produce RNA molecules that interfere with genes containing identical sequences.

**Results:**

CRISPR spacer #1, which matches a sequence within *hisS*, the histidyl-tRNA synthetase gene of *P. carbinolicus*, was shown to be expressed. Phylogenetic analysis and genetics demonstrated that a gene paralogous to *hisS *in the genomes of *Geobacteraceae *is unlikely to compensate for interference with *hisS*. Spacer #1 inhibited growth of a transgenic strain of *Geobacter sulfurreducens *in which the native *hisS *was replaced with that of *P. carbinolicus*. The prediction that interference with *hisS *would result in an attenuated histidyl-tRNA pool insufficient for translation of proteins with multiple closely spaced histidines, predisposing them to mutation and elimination during evolution, was investigated by comparative genomics of *P. carbinolicus *and related species. Several ancestral genes with high histidine demand have been lost or modified in the *P. carbinolicus *lineage, providing an explanation for its physiological differences from other *Geobacteraceae*.

**Conclusions:**

The disappearance of multiheme *c*-type cytochromes and other genes typical of a metal-respiring ancestor from the *P. carbinolicus *lineage may be the consequence of spacer #1 interfering with *hisS*, a condition that can be reproduced in a heterologous host. This is the first successful co-introduction of an active CRISPR spacer and its target in the same cell, the first application of a chimeric CRISPR construct consisting of a spacer from one species in the context of repeats of another species, and the first report of a potential impact of CRISPR on genome-scale evolution by interference with an essential gene.

## Background

Clustered regularly interspaced short palindromic repeats (CRISPR), which consist of direct repeats of a short sequence (21-47 bp) separated by nonrepetitive sequences of similar size, have been identified in the genome sequences of almost all archaea and numerous bacteria, with a variable complement of adjacent CRISPR-associated (*cas*) genes [1-9]. A fraction of the spacer sequences between repeats have been found to match sequences termed "proto-spacers" within genes, from which they may be derived [8,10,11], and the fact that many of these genes belong to phage or plasmid entities led to the hypothesis that CRISPR and the Cas proteins may function as an RNA interference-based immune system [6]. The link between specific CRISPR spacers and proto-spacers and phage resistance has been established by mutational analysis in *Streptococcus thermophilus *[12,13], and by testing synthetic CRISPR constructs in *Escherichia coli *[14]. Similarly, resistance of *Staphylococcus epidermidis *to a conjugative plasmid has been shown to depend on a CRISPR spacer and the corresponding proto-spacer [15]. Expression of CRISPR loci as long transcripts processed into smaller RNA molecules has been observed in several archaea [5,16-19] and bacteria [14,20]. A complex of Cas proteins has been shown to carry out this processing in *E. coli *and to be required for resistance to infection [14]; a different protein (Cas6) processes CRISPR transcripts in *Pyrococcus furiosus *[21]. CRISPR-derived RNAs have been shown to form RNA-protein complexes in *P. furiosus *[19], which leads to degradation of RNAs containing matching proto-spacers [22], whereas DNA was shown to be the target of interference by spacer-containing RNAs in *S. epidermidis *[15]. Although CRISPR are widely regarded as an immunological phenomenon, CRISPR and *cas *genes have also been implicated in spore development of *Myxococcus xanthus *[3,23] and in inhibition of biofilm formation and swarming of *Pseudomonas aeruginosa *by a lysogenic phage [24], and there has been speculation that spacers with matches to housekeeping genes represent a novel mechanism of gene regulation [25].

The *Geobacteraceae*, a Fe(III)-respiring family of *Deltaproteobacteria*, are of interest for their role in bioremediation of U(VI)-contaminated environments and their ability to donate electrons directly to graphite electrodes, producing an electrical current [26,27]. *Pelobacter carbinolicus *is a member of the *Geobacteraceae *that grows by fermentation of acetoin and 2,3-butanediol, as well as by indirect Fe(III) respiration with ethanol as the electron donor and acetate as the end product [28,29]. Unlike its relatives in the genus *Geobacter*, *P. carbinolicus *cannot reduce Fe(III) directly in the absence of sulfur or sulfide [30], or produce electricity [31]. The genome of *P. carbinolicus *was sequenced for the purpose of comparison to those of *Geobacter *species, three of which have been extensively curated: *Geobacter sulfurreducens *[32], *Geobacter metallireducens *[33] and *Geobacter bemidjiensis *(Aklujkar *et al*., submitted). This report explores how evolution of the *P. carbinolicus *genome may have been influenced by a spacer within the CRISPR locus that matches a proto-spacer within histidyl-tRNA synthetase (*hisS*), resulting in the elimination of ancestral genes containing multiple closely spaced histidines. The interfering nature of the spacer was confirmed by introducing it to a transgenic *G. sulfurreducens *strain containing the target gene.

## Methods

### Analysis of CRISPR spacers

The CRISPR locus was identified when manual curation of the *P. carbinolicus *genome revealed a series of suspiciously repetitive predicted genes. The nonredundant nucleotide sequence database was queried with each of the 111 CRISPR spacers of *P. carbinolicus *using the BLAST algorithm [34], with the minimum possible word size of 7 bp and without filtering out low-complexity regions of the queries. Alignments with five or fewer mismatches out of 32 bases were considered significant.

### Phylogenetic analysis of HisS and HisZ proteins

The sequences of all predicted *hisS *gene products of the *Geobacteraceae*, together with HisS and HisZ protein sequences representative of various families of Bacteria and Archaea, were aligned by TCoffee [35] and trimmed using Mesquite (Maddison, W. P., and Maddison, D. R.. 2006. Mesquite: a modular system for evolutionary analysis. Version 1.12). Phylogenetic trees were constructed using Phylip (Felsenstein, J. 2005. PHYLIP (Phylogeny Inference Package) version 3.6) with 500 bootstrap runs.

### Quantitative real-time PCR of reverse-transcribed RNA

*P. carbinolicus *strain DSM2380 was grown as previously described [36] with ethanol as the electron donor and Fe(III) as the electron acceptor. RNA was isolated from triplicate chemostat cultures as previously described [37,38]. Transgenic *G. sulfurreducens *strains were grown in NBAF medium [39] and RNA was isolated from actively growing triplicate batch cultures at an OD_600 _of 0.20 to 0.31. The absence of DNA contamination was confirmed by PCR as previously described [36] with primer pairs specific for CRISPR spacer #1, for *hisS *and for *hisZ *(Table 1), using *P. carbinolicus *or *G. sulfurreducens *genomic DNA (isolated with the MasterPure DNA Purification Kit from EPICENTRE Biotechnologies, Madison, WI) as a control. Six to twelve clones of each genomic DNA PCR product were sequenced to verify the specificity of the primers. Reverse transcription was performed with the Enhanced Avian First Strand Synthesis Kit (Sigma-Aldrich, St. Louis, MO) as described previously [37], using each primer individually at 2 μM concentration with 400 ng of RNA in 20 μl total volume. Successful reverse transcription and the feasibility of DNA amplification in the presence of RNA were verified by PCR using 5 μl of this reaction. Quantitative real-time PCR (QRT-PCR) was performed with two to four technical replicates (9.5 μl of a tenfold dilution of cDNA, corresponding to 19 ng of RNA) for each of three biological replicates in a Taqman 7500 instrument using 2 × Power SYBR Green PCR master mix (Applied Biosystems, Foster City, CA) and primer pairs at 9 nM concentration in 25 μl total volume, for 50 cycles with an annealing temperature of 60°C and triplicate standards of spacer #1 and *hisS *PCR products from *P. carbinolicus *genomic DNA and a *hisZ *PCR product from *G. sulfurreducens *genomic DNA, encompassing four orders of magnitude.

**Table 1:** Oligonucleotides for QRT-PCR and genetic manipulations.

Primers for QRT-PCR			
Name	Purpose	Location	Sequence (5' to 3')
MA0326		spacer #2	CCTGGTTGAGGTTAGCGTTGA
	PCR of spacer #1		
MA0327		outside CRISPR	AATTCGGTGGCCAGTTGTTC

MA0328		sense strand	CAGGAAGCCACCAAGGAT
	PCR of *hisS * Pcar_1041		
MA0329		antisense strand	TGGGAGCCGAGTTGATTG

MA0441		sense strand	CAAACTGATTGCCGTTCCTT
	PCR of *hisZ * GSU3307		
MA0442		antisense strand	AGGCCGATGAGTTCTACGC

**Primers for construction of *hisS *transgenic strain MA159**			

**Name**	**Purpose**	**Sequence (5' to 3')**	

MA0330		TGACATCTCGCTGGACCGGG	
	PCR on 5' side of *hisS * GSU1659		
MA0331		CTATGCTAGCACTAGTTTGTAATCATGAACGTACCTACTC CTTTAATTG	

MA0332		GTACGTTCATGATTACAAACTAGTGCTAGCATAGCAATAC CTGCATTG	
	PCR on 3' side of *hisS* GSU1659		
MA0333		AGTCCATTCCTCCTGTGG	

MA0334		AAGGGATCTATCATGAGCATATCAGGCATTAAGGG	
	PCR of *hisS * Pcar_1041		
MA0335		GCGCGGCGCGACTAGTTTCCTCGTGTCTTTTCC	

MA0052		TGCATATGGCTCTAGAATAACTTCGTATAGC	
	gentamicin marker		
MA0053		TCGATAAGCTTCTAGAATAACTTCGTATAATG	

**Oligonucleotides for construction of chimeric CRISPR expression plasmid pMA35**			

**Name**	**Purpose**	**Sequence (5' to 3')**	

MA0269		ACATGTCACTGCCCGCTTTCCAGTC	
	PCR of *lacI*- *taclacUV5 * promoter		
MA0270		GCATGCGTGTGAAATTGTTATCCGC	

MA0429		AATTCGGTTCATCCCCGCGCATGCGGGGAACACATACAT GAGGGCAAACGCCTTTTGGCCGGCGGCGGTTCATCCCCG CGCATGCGGGGAACACG	
	synthetic CRISPR of spacer #1		
MA0430		GATCCGTGTTCCCCGCATGCGCGGGGATGAACCGCCGCC GGCCAAAAGGCGTTTGCCCTCATGTATGTGTTCCCCGCAT GCGCGGGGATGAACCG	

MA36R	sequencing	CGACATCATAACGGTTC	

Note: Within the sequence of the chimeric CRISPR, a single base pair (underlined) has been duplicated in plasmid pMA35-!, at the exact centre of spacer #1.

### Recombinant DNA techniques

All restriction enzymes were purchased from New England Biolabs; LA Taq polymerase was from Takara Mirus Bio; plasmids were propagated in *E. coli *TOP10 cells from Invitrogen; DNA purification kits for plasmids and agarose gel slices were from QIAGEN, and the MasterPure kit for genomic DNA extraction was from EPICENTRE. To construct a transgenic strain of *G. sulfurreducens *in which the native *hisS *gene was replaced with *hisS *from *P. carbinolicus*, three primer pairs (Table 1) were used to amplify the 5'-side and 3'-side flanking regions of *hisS *GSU1659 and the coding sequence of *hisS *Pcar_1041. The two flanking regions were digested with *Spe *I and ligated; this product and the Pcar_1041 amplicon were separately TOPO-cloned (Invitrogen) and sequenced. Digestion with *Bsp*H I (overlapping the start codon) and *Spe *I (overlapping the stop codon) was used to insert the Pcar_1041 gene between the flanking regions of GSU1659. As a selectable marker, a gentamicin resistance cartridge was amplified from plasmid pCM351 [40] with *Xba *I site-containing primers (Table 1), maintained as a TOPO clone, and ligated into the *Nhe *I site between the *Spe *I site and the 3' flanking region of GSU1659. (A similar construct, in which only the marker was inserted without Pcar_1041, was used in unsuccessful attempts to delete GSU1659.) The entire *hisS *replacement construct was excised using *Eco*R I, purified from an agarose gel, and electroporated into the wild type *G. sulfurreducens *strain DL1 as previously described [39]. An isolated gentamicin-resistant colony was streaked for purity before transfer to liquid. The genotype of this strain, called MA159G, was confirmed by PCR of genomic DNA, with primers MA0334 and MA0335 (which amplify *hisS *Pcar_1041 but not *hisS *GSU1659) as well as MA0330 and MA0333 (which give a larger product for MA159G than for DL1, due to the inserted marker). The marker, which had *loxP *sites on either side, was removed from the chromosome of strain MA159G by introducing the Cre recombinase expression plasmid pCM158 [40] by electroporation and selecting for resistance to kanamycin (Sigma). Four colonies of the resultant strain called MA159 were streaked for purity and their genotypes were confirmed by PCR; amplicons were digested with *Pst *I to distinguish Pcar_1041 from GSU1659. This strain was electroporated with a plasmid called pRG6 (R. Glaven, personal communication), which is incompatible with pCM158 and selectable with spectinomycin (Sigma); it differs from pRG5 [41] only in that it carries the *lacI *repressor gene. The chromosomal genotype of this strain was confirmed by PCR and *Pst *I digestion.

A plasmid vector called pMA36, incompatible with pRG6, was constructed for isopropylthio-β-D-galactopyranoside (IPTG)-inducible expression of a chimeric CRISPR containing spacer #1 from *P. carbinolicus *between two repeats typical of the CRISPR2 locus of *G. sulfurreducens*. The *lacI *repressor gene and *taclacUV5 *promoter of plasmid pCD341 [42] were amplified by PCR with *Pci *I and *Sph *I site-containing primers (Table 1), TOPO-cloned and sequenced, and excised for ligation into plasmid pCM66 [43], resulting in plasmid pMA36. The chimeric CRISPR, consisting of annealed oligonucleotides MA0429 and MA0430 (Table 1), was ligated between the *Bam*H I and *Eco*R I sites of pMA36. The sequence of this plasmid, called pMA35-1, was confirmed using the sequencing primer MA36R (Table 1). Serendipitously, two variants were discovered: pMA35-2 in which the chimeric CRISPR had expanded to two copies of spacer #1 with a third copy of the repeat between them, and pMA35-! in which spacer #1 was disrupted by duplication of a single G:C base pair at its exact centre (underlined in Table 1). All three chimeric CRISPR expression plasmids were electroporated into DL1 and MA159(pRG6), in parallel with pMA36 as a control. The genotypes of multiple kanamycin-resistant colonies of each transformation were confirmed by PCR of Pcar_1041 followed by *Pst *I digestion as well as cloning and sequencing, and by sequencing of plasmids present in the genomic DNA extracts (after transformation into *E. coli *to improve DNA quality). Another variant of pMA35-1 called pMA35-0 was serendipitously discovered in which spacer #1 had been deleted by recombination of the repeats on either side.

### Growth conditions

Transformants of *G. sulfurreducens *were selected on NBAF medium [39] containing 1.5% Agar Noble (Difco), supplemented with 5 mM cysteine hydrochloride and 0.1% yeast extract in an anaerobic chamber. Growth experiments were carried out with liquid cultures in either NBAF medium supplemented with 1 mM cysteine hydrochloride or FWAFC medium [39] modified to contain 10 mM acetate and supplemented with 1 mM ferrous ammonium sulfate, in an atmosphere of N_2 _and CO_2 _(80%:20%) in rubber-stoppered 26 ml glass tubes at 30°C.

### Bioinformatics

Codon usage was determined using the CodonFrequency algorithm of the Genetics Computer Group Wisconsin Package version 10.3 (Accelrys Inc., San Diego, CA). A script to compute the number of histidines and the distances between them for every protein sequence in a list was written in Perl.

## Results

### The CRISPR locus of *P. carbinolicus *includes a spacer matching its own histidyl-tRNA synthetase

**Figure 1:**
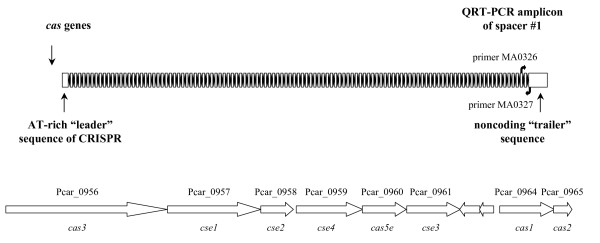
**The CRISPR locus of *P. carbinolicus***. This locus consists of 112 repeats (black diamonds) separated by 111 nonrepetitive spacers (white rectangles). Spacer #1 is at the trailer end, farthest from the *cas *genes and the AT-rich leader sequence near which new spacers are typically inserted. Primers MA0326 and MA0327 are based on sequences surrounding spacer #1, and were used to detect its RNA transcript. The arrangement of the *cas *genes (located immediately to the left of the leader sequence) is illustrated in the lower half of the figure. The two intervening genes encode a putative toxin (Pcar_0962) and transcriptional regulator or antitoxin (Pcar_0963).

**Figure 2:**
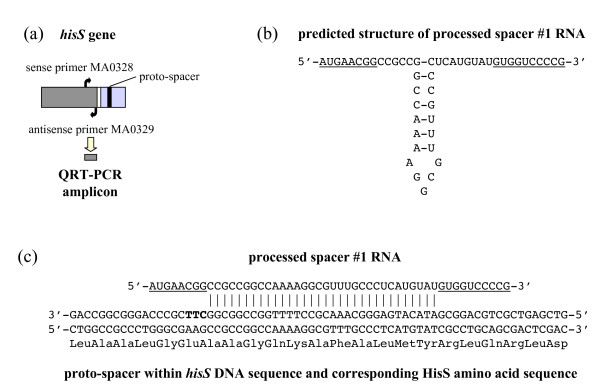
**CRISPR spacer #1 matches a nucleotide sequence within the *hisS *gene**. (a) *hisS *consists of a catalytic domain (dark grey) and an anticodon loop recognition domain (light grey) connected by a linker (white stripe). The proto-spacer sequence matching spacer #1 (black stripe) is within the anticodon loop recognition domain. Primers MA0328 and MA0329 were designed to amplify a cDNA segment from the catalytic domain. (b) Predicted secondary structure of a processed CRISPR transcript (initiated at the leader sequence) that contains spacer #1, before hybridization to *hisS *DNA. Sequences from the repeats flanking spacer #1 are underlined. (c) Predicted hybridization of a proto-spacer segment within the anticodon loop recognition domain of *hisS *DNA (template strand) with a processed spacer #1 RNA. The proto-spacer-adjacent motif CTT is shown in bold.

During manual curation of the *P. carbinolicus *genome annotation, the CRISPR locus was identified as 112 repeats of the sequence 5'-GAGT**TCCCCGCA**GA**TGCGGGGA**TGAACCG-3' (bases in bold predicted to form a hairpin), separated by spacer sequences of 32 bp (Figure 1). This repeat sequence belongs to phylogenetic cluster 2 of the CRISPR classification system [44] and the adjacent *cas *genes (Figure 1) are of the subtype "Ecoli" [3]. The nonredundant nucleotide sequence database was queried in an attempt to identify genes from which the 111 CRISPR spacers of *P. carbinolicus *might be derived. The only hits with five or fewer mismatched bases were hits with zero mismatches within the *P. carbinolicus *genome itself: spacer #1, located at the "trailer" end of the locus, farthest from the AT-rich "leader sequence" and *cas *genes encoding CRISPR-associated proteins (Figure 1), matched a sequence within the histidyl-tRNA synthetase (*hisS*) gene Pcar_1041 (Figure 2); spacer #43 matched the adjacent spacer #44; and spacer #28 matched the nonadjacent spacer #50. Spacer #1 is likely to be the oldest spacer because new spacers are added next to the leader sequence upon exposure of streptococci to bacteriophage [12,13,45-47], and closely related strains of bacteria and archaea contain identical spacers only near the trailer ends of their CRISPR [2,5,11,18,45,48-51]. This observation led to the hypothesis that *P. carbinolicus *has experienced interference with the *hisS *gene, encoding an essential housekeeping enzyme, over a significant period of its evolutionary history.

### Quantitative detection of CRISPR spacer #1 transcripts

**Figure 3:**
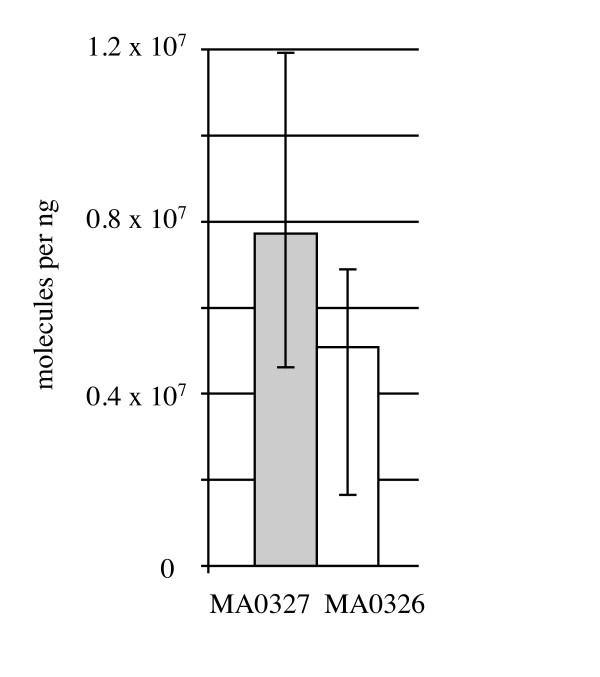
**Spacer #1 is transcribed into RNA in *P. carbinolicus*, with both strands similarly abundant**. Reverse transcription was performed with either primer MA0327 (grey bar) or primer MA0326 (white bar), and the amount of cDNA was quantified by QRT-PCR. The mean of three biological replicates is shown; error bars represent the minimum and maximum.

In an attempt to determine whether spacer #1 is transcribed into RNA that could have interfered with the *hisS *gene at one time, and which strand of trailer end RNA is predominant in *P. carbinolicus*, two oligonucleotide primers were designed flanking spacer #1 (Figure 1, Table 1): MA0326 within spacer #2 and MA0327 just outside the outermost repeat of the CRISPR. Reverse transcription of *P. carbinolicus *RNA into cDNA was attempted with each single primer, followed by quantitative real-time PCR amplification with both primers. The amount of spacer #1-containing RNA including the sequence found on the sense strand of *hisS*, detected by primer MA0327, was not significantly different from the amount of spacer #1-containing RNA corresponding to the antisense of *hisS*, detected by primer MA0326 (Figure 3). Control PCR reactions without reverse transcription yielded no product, indicating that DNA contamination was negligible and only RNA of both strands was detected.

The sense strand spacer #1-containing RNA detected in this experiment may represent the 3' end of a long transcript initiated near the leader sequence, whereas the antisense strand spacer #1-containing RNA may be produced independently from a promoter at the opposite end of the CRISPR. It is also possible that one strand is produced from the other by an unidentified RNA-directed RNA polymerase. If the sense strand spacer #1-containing RNA undergoes processing similarly to the *E. coli *CRISPR transcript [14], which belongs to the same phylogenetic cluster of repeat sequences as the *P. carbinolicus *CRISPR [44], cleavage within the stem-loops of the repeats flanking spacer #1, followed by 3' end trimming, would release a short RNA with predicted secondary structure (Figure 2b). This or the corresponding antisense strand spacer #1-containing RNA may hybridize to the proto-spacer DNA sequence within the *hisS *gene (Figure 2c).

### Phylogenetic and experimental evidence that interference with *hisS *cannot be compensated

It is surprising that spacer #1 is retained by *P. carbinolicus *if it interferes with the essential function of histidine activation for protein synthesis. Comparative genome analysis revealed that *P. carbinolicus *and its close relatives, the *Geobacteraceae*, possess two full-length *hisS*-like genes, whereas other bacteria have only one. Interference with Pcar_1041 by spacer #1 might have had negligible effect if Pcar_0202 also produced histidyl-tRNA synthetase activity. However, both phylogenetic and mutational studies suggest that Pcar_1041 is essential, being the only real *hisS*, as detailed below.

**Figure 4:**
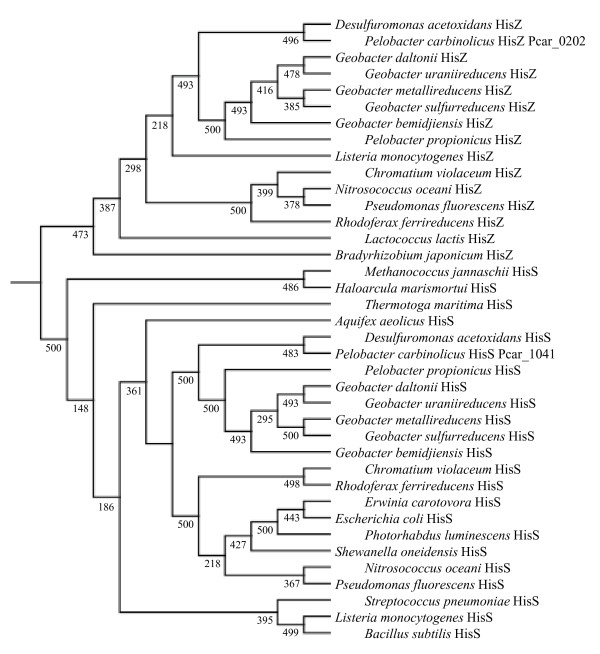
**Phylogeny of HisS and HisZ proteins**. Pcar_1041 and orthologous proteins of *Geobacteraceae *cluster among true histidyl-tRNA synthetases (HisS), whereas Pcar_0202 and its orthologs cluster among the HisZ proteins, which are the regulatory subunit of ATP phosphoribosyltransferase. Confidence values are out of 500 bootstraps.

In some bacteria there is a *hisS*-related gene called *hisZ*, which produces a protein that lacks the C-terminal anticodon loop recognition domain of a true histidyl-tRNA synthetase, functioning instead as a regulatory subunit of ATP phosphoribosyltransferase, the first enzyme of histidine biosynthesis [52]. In bacteria that possess *hisZ*, the *hisG *gene encoding the catalytic domain of ATP phosphoribosyltransferase is shorter than in bacteria that do not possess *hisZ *[53]. A short *hisG *gene is present in *P. carbinolicus *and all other *Geobacteraceae*, but unlike previously described *hisZ *genes, both *hisS*-like genes contain obvious anticodon loop recognition domains. Phylogenetic analysis showed that the Pcar_1041 gene product and orthologous protein sequences of *Geobacteraceae *cluster among the HisS proteins of other bacteria, whereas the Pcar_0202 gene product and its orthologs belong among the HisZ proteins (Figure 4). Furthermore, the ortholog of Pcar_0202 in *G. sulfurreducens *(GSU3307) could be deleted (Aklujkar and Lovley, manuscript in preparation), whereas the ortholog of Pcar_1041 (GSU1659) could only be replaced with Pcar_1041 (this study). Three electroporation attempts failed to delete GSU1659 outright. This result indicates that Pcar_0202 and its orthologs lack significant histidyl-tRNA synthetase activity, and suggests that interference with Pcar_1041 by spacer #1 would exert severe pressure on *P. carbinolicus*.

The phylogenetic tree also demonstrates that the *hisS *gene Pcar_1041 was not acquired laterally; it is clearly an ancestral gene containing a proto-spacer that is not present in its closest relatives.

### Spacer #1 inhibits growth of a transgenic *G. sulfurreducens *strain containing *hisS *of *P. carbinolicus*

**Figure 5:**
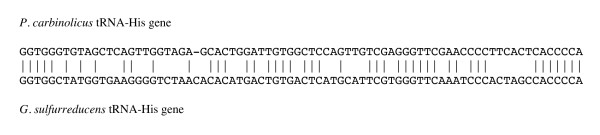
**Alignment of the sequences of tRNA-His genes from *P. carbinolicus *and *G. sulfurreducens***.

**Figure 6:**
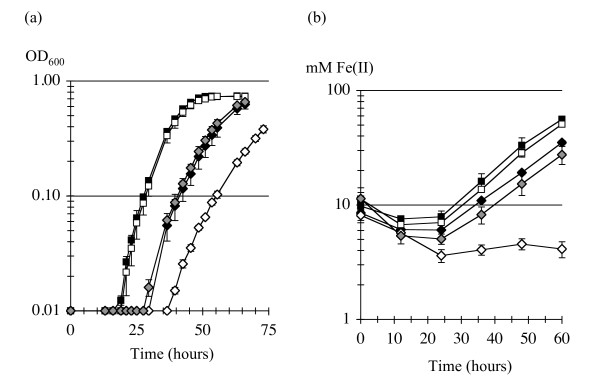
**Growth of *G. sulfurreducens *with *hisS *of *P. carbinolicus *is inhibited by spacer #1**. (a) Growth on NBAF medium by reduction of fumarate. (b) Growth on FWAFC medium by reduction of Fe(III) citrate. The strains shown are wild type *G. sulfurreducens *DL1 (black squares); DL1(pMA35-2) with two copies of spacer #1 in a plasmid-borne chimeric CRISPR (white squares); transgenic strain MA159, which has *hisS *of *P. carbinolicus *(black diamonds); MA159(pMA35-2) with both the *hisS *transgene and two copies of spacer #1 (white diamonds); and MA159(pMA35-0) with the *hisS *transgene and a CRISPR repeat without spacer #1 (grey diamonds).

It is not yet possible to make mutations in *P. carbinolicus*. Therefore, interference of spacer #1 with *hisS *was tested in the more genetically tractable species *G. sulfurreducens*, in which the repeat sequence of the CRISPR2 locus (5'-GTGT**TCCCCGC**ATGC**GCGGGGA**TGAACCG-3') is very similar to that of the *P. carbinolicus *CRISPR. A plasmid called pMA35-1 was designed for IPTG-inducible expression of a chimeric CRISPR construct consisting of spacer #1 of *P. carbinolicus *between two copies of the *G. sulfurreducens *repeat, and a transgenic strain of *G. sulfurreducens *called MA159 was generated in which the native *hisS *gene GSU1659 was replaced by Pcar_1041, the *hisS *gene of *P. carbinolicus*. The tRNA-His sequences of the two species are very different (Figure 5), suggesting that the histidyl-tRNA synthetase of *P. carbinolicus *might have difficulty recognizing its substrate in *G. sulfurreducens*. However, replacement of GSU1659 with Pcar_1041 resulted in a viable strain, which grew more slowly than the wild type (Figure 6).

Despite prior expression of the LacI repressor protein from plasmid pRG6 to prevent premature expression of the chimeric CRISPR, electroporations of MA159(pRG6) with pMA35-1 and two serendipitously obtained variants (pMA35-2 with two copies of spacer #1 and pMA35-! with spacer #1 interrupted by a single base pair insertion as shown in Table 1) were marginally successful (yielding zero to 4 colonies per attempt), whereas electroporation with an equal amount of the empty vector pMA36 produced hundreds of colonies per attempt. Electroporations of the wild type *G. sulfurreducens *strain DL1, carried out in parallel, yielded hundreds of colonies for all three chimeric CRISPR expression plasmids. These observations suggest that even leaky expression of the chimeric CRISPR containing spacer #1, or its mere presence as DNA, is largely incompatible with the *hisS *gene of *P. carbinolicus *containing the matching proto-spacer, which is present in the MA159 host, but not with the native *hisS *gene of *G. sulfurreducens *in the DL1 host.

Growth experiments provided further proof that spacer #1 interferes with *hisS *of *P. carbinolicus*. The *G. sulfurreducens *transformants were first checked by PCR, restriction digestion, and sequencing to verify that the *hisS *transgene and spacer #1 were intact. In one transformant, spontaneous recombination of the repeats on either side of spacer #1, eliminating it from the chimeric CRISPR expression plasmid, resulted in a strain called MA159(pMA35-0) that possessed both *hisS *of *P. carbinolicus *and the repeat sequence on the plasmid, but no spacer #1. Compared to this control that grew similarly to MA159, the presence of spacer #1 in the other MA159 transformants (i.e., with *hisS *of *P. carbinolicus*) resulted in long lag periods and somewhat reduced growth rates in NBAF medium with fumarate as the electron acceptor (Figure 6a), and very poor growth in FWAFC medium with Fe(III) citrate as the electron acceptor (Figure 6b). This effect was the same with either one copy of spacer #1 in MA159(pMA35-1) or two copies in MA159(pMA35-2), or with a single base pair insertion in spacer #1 in MA159(pMA35-!) - for the sake of clarity, only MA159(pMA35-2) is shown. The only exception was that in one experiment, triplicate cultures of MA159(pMA35-!) grew especially poorly after three transfers in NBAF (not shown). Wild type growth patterns were observed when any of the three plasmids was present in the DL1 host (i.e., with *hisS *of *G. sulfurreducens*) - for clarity, only DL1(pMA35-2) is shown (Figure 6). Although expression of spacer #1 is expected to be low in the absence of IPTG, growth inhibition of the MA159 strains was observed, and addition of IPTG had no effect, indicating that expression of chimeric CRISPR RNA was not the limiting factor for inhibition of growth.

### Spacer #1 reduces the amount of *hisS *RNA in transgenic *G. sulfurreducens *no more than it affects *hisZ *RNA

**Figure 7:**
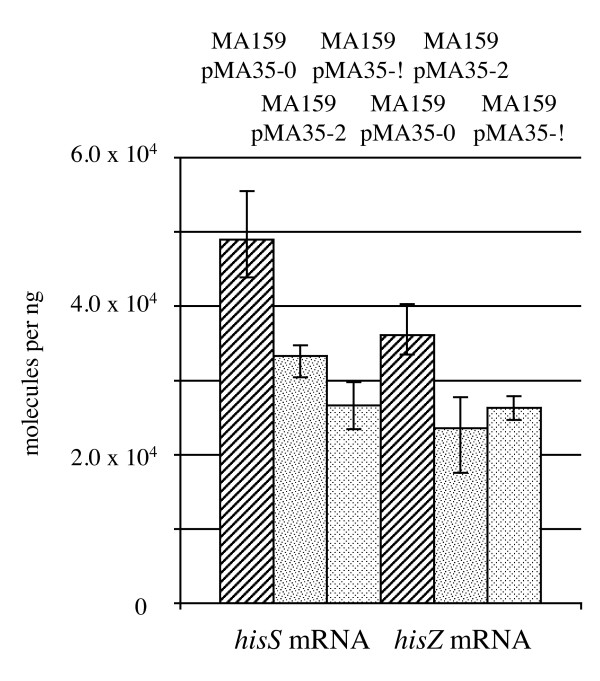
**Spacer #1 has similar effects on the amounts of *hisS *and *hisZ *RNA**. The strains shown are *G. sulfurreducens *MA159(pMA35-0) with the *hisS *transgene and a CRISPR repeat without spacer #1 (diagonally striped bars); MA159(pMA35-2) with both the *hisS *transgene and two copies of spacer #1 (speckled bars); and MA159(pMA35-!) with the *hisS *transgene and a single mutated copy of spacer #1 (diamond-patterned bars). Reverse transcription was performed with either primer MA0329 for *hisS *or primer MA0442 for *hisZ*, and the amount of cDNA was quantified by QRT-PCR. The mean of three biological replicates is shown; error bars represent the minimum and maximum.

Total RNA was isolated from NBAF-grown cultures of strains containing *hisS *of *P. carbinolicus*. The amount of *hisS *RNA was higher in the control MA159(pMA35-0) strain than in the growth-inhibited MA159(pMA35-2) strain with spacer #1 (Figure 7), and lowest in the MA159(pMA35-!) strain that had the most severe growth defect in a parallel growth experiment using the same inoculum. However, when the amount of *hisZ *RNA was compared across the same three strains as a control, a similar pattern was observed (Figure 7), suggesting that reduced expression of other housekeeping genes besides *hisS *occurs when growth is slowed by the incompatibility between spacer #1 and *hisS*.

### The *P. carbinolicus *genome has fewer genes with numerous or closely spaced histidine codons than closely related genomes

**Figure 8:**
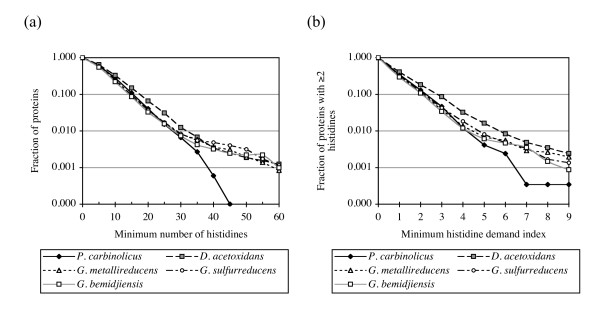
**The *P*. carbinolicus genome encodes fewer proteins with multiple closely spaced histidines**. (a) The fraction of proteins with a given minimum number of histidines, plotted for the genomes of *P. carbinolicus *(black diamonds), *D. acetoxidans *(grey squares), *G. sulfurreducens *(white circles), *G. metallireducens *(white triangles) and *G. bemidjiensis *(white squares). (b) The fraction of proteins with two or more histidines and a given minimum histidine demand index, plotted for the same five genomes.

The evidence that *P. carbinolicus *expresses CRISPR spacer #1, and that spacer #1 inhibits growth of a *G. sulfurreducens *strain that is dependent on *hisS *of *P. carbinolicus*, led to the question of whether any effect of this interference during recent evolution could be discerned in the genome of *P. carbinolicus*. If the expected shortage of histidyl-tRNA were occasionally severe enough for ribosomes to stall during translation of genes with numerous histidine codons, one would expect these genes to be predisposed for elimination from the genome, because abortive expression wastes energy and because any selective advantage of the genes would be diminished. Missense mutations of closely spaced histidine codons would also be favoured as long as they did not interfere with an essential function. Therefore, the number of histidine codons in every gene and the harmonic mean distance between histidine codons in every gene were computed for *P. carbinolicus *and its closest relative with a nearly completely sequenced genome, *Desulfuromonas acetoxidans *[GenBank:NZ_AAEW00000000], as well as for the completely sequenced and manually curated genome annotations of the more distantly related *G. sulfurreducens *[32], *G. metallireducens *[33] and *G. bemidjiensis *(Aklujkar *et al*., submitted). A plot of the fraction of protein sequences in each genome that have a given minimum number of histidines shows that the *P. carbinolicus *genome is deficient in genes with 35 or more histidine codons, and possesses none with 45 or more (Figure 8a). To identify ancestral genes that might have been counterselected in *P. carbinolicus *due to close spacing of histidine codons, an index of histidine demand was computed as the number of histidine codons in a gene divided by the harmonic mean distance between them. Fewer genes with histidine demand above 5.0 are present in the *P. carbinolicus *genome, and none has an index above 10.0 (Figure 8b). Despite these trends, the overall frequency of histidine codons in the *P. carbinolicus *genome is 22.50 per thousand, very similar to *D. acetoxidans *(23.94 per thousand), *G. sulfurreducens *(20.55 per thousand), *G. metallireducens *(20.42 per thousand) and *G. bemidjiensis *(19.76 per thousand). This observation is consistent with the expected effect of an acute histidyl-tRNA shortage in the vicinity of gene transcripts with multiple or closely spaced histidine codons undergoing translation, whereas a defect in histidine biosynthesis prior to histidyl-tRNA synthetase would be expected to affect histidine codon usage in general.

### The *P. carbinolicus *genome has lost ancestral genes with numerous or closely spaced histidines

Genes of *D. acetoxidans*, *G. sulfurreducens*, *G. metallireducens *and *G. bemidjiensis *that contain 35 or more histidine codons, or have a histidine demand index above 5.0, were examined in order to identify ancestral genes that have reduced their histidine demand or have been lost specifically in the *P. carbinolicus *genome (Additional file 1: Table S1). Many genes found in *Geobacter *species are not necessarily ancestral to *P. carbinolicus*; they lack homologs in either the unfinished *D. acetoxidans *genome or the partial genome sequences of a mixture of *D. acetoxidans *and *D. palmitatis *(D. R. L. and coworkers, unpublished). Other genes that are present in *D. acetoxidans*, but not *Geobacter *species, could have been acquired after divergence from *P. carbinolicus*. Five gene families actually show increased histidine demand in *P. carbinolicus *compared to other *Geobacteraceae*, and in many other cases, a *P. carbinolicus *gene has lower histidine demand than its orthologs, but is still above the cutoff value of 5.0, or contains a similar number of histidine codons. However, after all these considerations there remain sixteen clearly ancestral gene families with typically high histidine demand that are missing or have reduced histidine demand specifically in *P. carbinolicus *(Additional file 1: Table S1). The functional annotations of these gene families are briefly described below.

The *nuoL-1 *gene family, with a high-histidine-demand representative in every *Geobacter *species and *D. acetoxidans*, encodes the L subunit of an NADH dehydrogenase I complex that *P. carbinolicus *has entirely lost. The NuoL protein is thought to have a proton-pumping function [54], for which the imidazole groups of its clustered histidines are well suited.

The *znuA *(or *zntC*) gene encodes the periplasmic protein of an ATP-binding cassette transporter, within which a cluster of histidines is thought to bind zinc with high affinity [55]. The *znuA *gene of *D. acetoxidans *has eight histidines in its putative metal-binding cluster, whereas its closest relative, the *P. carbinolicus znuA *gene (Pcar_3026), has only four.

*D. acetoxidans *possesses two clearly ancestral genes related to arsenite *S*-adenosylmethyltransferase (Dace_2134, Dace_3081), with homologs in *Geobacter lovleyi *and other *Deltaproteobacteria *that contain numerous histidines. The closest homolog of Dace_3081 is in *P. carbinolicus *(Pcar_2089), with fewer histidines and a much reduced histidine demand index. *P. carbinolicus *lacks an ortholog of Dace_2134.

Two ancestral genes encoding polyketide synthase-type enzymes, with representatives in *D. acetoxidans*, *G. lovleyi*, *Geobacter uraniireducens*, *Geobacter bemidjiensis*, *Geobacter *sp. M21, *Geobacter *sp. M18, *Geobacter daltonii*, and *Pelobacter propionicus *that contain numerous histidines, have clearly been lost by *P. carbinolicus*. Both polyketide synthases are composed of unusual domain combinations. Dace_0979 and its orthologs contain just one elongation domain, one acyl carrier protein acylation domain, two to five acyl carrier protein domains, one reductase domain, and no dehydratase or thioesterase domains, suggesting that they build up a long-chain fatty acid with multiple hydroxyl groups, or possibly a storage polymer of a precursor resembling 3-hydroxybutanoate. Dace_1838 and its orthologs contain two elongation domains, one acyl carrier protein acylation domain, three to four dehydratase domains, and no acyl carrier protein or reductase or thioesterase domains, suggesting that their product may be polyhydroxylated, polyunsaturated, or cyclic. The loss of these two enzymes likely means that *P. carbinolicus *does not make secondary metabolites that are present in most other *Geobacteraceae*.

Three other high-histidine-demand gene families that *P. carbinolicus *has lost encode a DUF323 domain-containing methyltransferase (Dace_1886) with homologs in *G. uraniireducens*, *G. bemidjiensis*, *Geobacter *sp. M21, and *Geobacter *sp. M18, a glycoside hydrolase (GSU2359) with homologs in *D. palmitatis*, *G. bemidjiensis*, *Geobacter *sp. M21, *Geobacter *sp. M18, *G. daltonii*, and *P. propionicus*, and a predicted *c*-type heme-binding, GAF domain-containing phosphohydrolase (GSU2622) with homologs in *D. palmitatis*, *G. metallireducens*, *G. uraniireducens*, *G. bemidjiensis*, *Geobacter *sp. M21, *Geobacter *sp. M18, and *G. daltonii*. The specific reactions catalyzed by these enzymes are not known.

The remaining eight ancestral gene families are multiheme *c*-type cytochromes represented in *D. acetoxidans *or *D. palmitatis *and one or both of *G. sulfurreducens *and *G. metallireducens*. At least one gene of each family was most probably inherited by *P. carbinolicus *from a common ancestor of the *Geobacteraceae*, and then lost. Several other cytochrome families, found in the *Geobacter *genomes but not the incomplete *D. acetoxidans *and *D. palmitatis *genomes, may have been inherited and lost by *P. carbinolicus*, but the evidence is inconclusive. Nevertheless, it is notable that there are no cytochromes among the eighteen proteins of *P. carbinolicus *that have either more than 35 histidines or a histidine demand index above 5.0 (Additional file 1: Table S1).

## Discussion

Although CRISPR spacers that match phage/plasmid genes have been shown to confer immunity against infection [12,15], and it is known that their mode of action is distinct from previously described mechanisms of phage resistance [13], the significant number of spacers that match host genes have not been investigated [25]. The activity of CRISPR spacers against genes encoding essential housekeeping enzymes has not been demonstrated before, nor have its consequences for genome-scale evolution been examined. The present study establishes that when spacer #1 of the *P. carbinolicus *CRISPR and its putative target, the *hisS *gene of the same species, are both present in a transgenic *G. sulfurreducens *strain, interference occurs that severely affects growth. Very few cells containing the *hisS *transgene were able to take up the chimeric CRISPR expression plasmids. Transformants in which spacer #1 was present grew more poorly. Attempts to induce expression of spacer #1 with IPTG had no effect, but growth was almost totally inhibited by switching from fumarate to Fe(III) as the electron acceptor, under which condition expression of Cas proteins and the chromosomal CRISPR2 is upregulated [56]. Therefore, the limiting factor for interference with *hisS *may be the amount of one or more Cas proteins, or the leader sequence, rather than the amount of spacer #1 transcript. Alternatively, growth by respiration of Fe(III) may require protein factors that cannot be expressed properly when histidyl-tRNA synthetase activity is low. Induction of CRISPR expression with IPTG in *S. epidermidis *has also shown no effect on interference with a target plasmid [15], but that CRISPR was inactive without a leader sequence in *cis*, whereas severe interference between spacer #1 and *hisS *requires neither the native leader sequence of the *G. sulfurreducens *CRISPR nor that of the *P. carbinolicus *CRISPR in *cis*.

The small decrease in the amount of *hisS *RNA in the presence of spacer #1, comparable to the decrease in the amount of transcript for a control housekeeping gene (*hisZ*) that accompanies the growth defect, is unlike the extent of decimation that one would expect if spacer #1-containing RNA catalyzed degradation of *hisS *RNA. It is even less likely that any transgenic strains could be viable if spacer #1-containing RNA targeted *hisS *DNA for degradation. An alternative possibility is that spacer #1 RNA hybridizes with the *hisS *gene without marking it for degradation (but perhaps recruiting one or more proteins), and must be displaced by RNA polymerase in order to complete transcription. In this scenario, even leaky expression of spacer #1 would saturate the available targets, and overexpression of spacer #1-containing RNA would not prevent RNA polymerase from displacing the one molecule obstructing transcription of the *hisS *gene. Consistent with these predictions, induction of spacer #1 expression with IPTG was not required for inhibition of growth, and did not cause stronger inhibition. It will be interesting to examine whether any putative nucleases have mutated in the few viable transformants that carry both spacer #1 and its target. None of the Cmr proteins implicated in RNA-targeted CRISPR function in *P. furiosus *[22] has a homolog in *G. sulfurreducens *or *P. carbinolicus*, nor does the CRISPR-associated double-stranded DNA/RNA-specific endonuclease of *Sulfolobus solfataricus *[57].

Sequencing confirmed that neither *hisS *nor spacer #1 was mutated in the viable transformants. Therefore, in contrast to earlier studies that showed absolute incompatibility between host spacers and phage proto-spacers, evaded only by mutation of the proto-spacers or proto-spacer-adjacent motifs [12,13], these results indicate that it is possible to establish a spacer that persistently interferes with an essential housekeeping gene. Both spacer and target can be maintained intact over numerous generations of inhibited growth. Insertion of a single base pair in the middle of the spacer did not reduce its efficacy, indicating that a small bulge in the region of complementarity between spacer and proto-spacer does not necessarily mitigate interference.

The experiments described herein with the chimeric CRISPR construct also show that a spacer from one species can be active in the context of the somewhat different repeats and *cas *genes of another species. The *P. carbinolicus *and *G. sulfurreducens *genomes encode homologs of all five components of the Cascade complex (*cse1 *CasA Pcar_0957, GSU1385; *cse2 *CasB Pcar_0958, GSU1386; *cse4 *CasC Pcar_0959, GSU1387; *cas5e *CasD Pcar_0960, GSU1388; and *cse3 *CasE Pcar_0961, GSU1389), which processes CRISPR transcripts into target-active RNAs in *E. coli *[14], along with the Cas3 helicase (Pcar_0956, GSU1384) that is required for their activity against targets [14], the Cas2 endoribonuclease (Pcar_0965, GSU1393) that may degrade them [58], and the Cas1 endodeoxyribonuclease (Pcar_0964, GSU1392) that may aid in the acquisition of new spacers [59], and promote DNA/RNA annealing [60]. However, the sequences of most of these Cas proteins are so divergent between the two species (e.g. 28% identity for Cas3) that it is noteworthy that the *G. sulfurreducens *system seemingly required no context other than its cognate repeat to recognize spacer #1 as a guide and *hisS *of *P. carbinolicus *as a target. There is evidence that proto-spacer-adjacent motifs are determinants of target recognition [13,20]. The proto-spacer within *hisS *is followed by a CTT motif (Figure 2), typical of sequences captured by CRISPR of phylogenetic cluster 2 [61], to which the *P. carbinolicus *CRISPR and *G. sulfurreducens *CRISPR2 loci both belong [44]. The facile reconstitution of interference between species with similar repeats and proto-spacer-adjacent motifs despite Cas protein divergence is encouraging for future development of CRISPR-based gene silencing technology with synthetic spacers.

**Figure 9:**
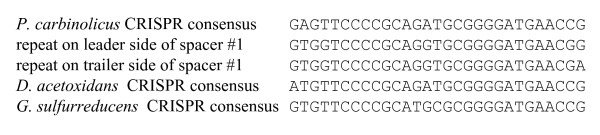
**Alignment of the repeats on either side of spacer #1 with the CRISPR consensus sequences of *P*. carbinolicus, *D. acetoxidans *and *G. sulfurreducens***.

A CRISPR transcript containing spacer #1 and flanking sequences is expressed in *P. carbinolicus*, and potentially processed by the Cas proteins into a *hisS*-interfering RNA. There are at least four possible explanations why the spacer and proto-spacer, which are incompatible in the transgenic *G. sulfurreducens *strain, still co-exist in *P. carbinolicus*. Firstly, being the spacer farthest from the leader sequence in *P. carbinolicus*, spacer #1 may produce comparatively few target-active RNA molecules, as processed RNAs containing spacers distal to the leader sequence are underrepresented in a clone library from *P. furiosus *[19]. Secondly, the repeats on either side of spacer #1 may not be good targets for processing because they deviate from the consensus repeat sequence of the *P. carbinolicus *CRISPR more than it has diverged from that of *D. acetoxidans *since the time of their last common ancestor (Figure 9). Thirdly, pairing of the two strands of RNA derived from spacer #1 might prevent targeting of the proto-spacer. The fourth possibility is that *P. carbinolicus *in its natural environment experiences only growth conditions under which the incompatibility between spacer #1 and *hisS *is permissive, as it is for the transgenic strain growing by respiration of fumarate, and has evolved to avoid growth conditions under which the incompatibility is absolute, comparable to respiration of Fe(III) by the transgenic strain.

Both strands of spacer #1 were detected at similar levels in *P. carbinolicus *RNA from actively growing cultures, in contrast with previous observations that both strands are present as RNA in *Sulfolobus acidocaldarius *only during stationary phase [5], that unequal amounts of RNA were detected with probes for the two strands in *P. furiosus *[19] and *Thermus thermophilus *[62], and that only transcripts from one strand were detected in *E. coli *[14], *S. epidermidis *[15], and *Xanthomonas oryzae *[20]. The presence of RNA representing both strands is consistent with CRISPR spacers on one strand of a cluster matching both the sense and antisense of proto-spacers [6], and with artificial spacers in both orientations having activity against their targets, although the efficacy may differ by several orders of magnitude [14].

Together, the evidence of spacer #1 expression and the proof that spacer #1 can inhibit growth in a *hisS*-dependent manner indicate that interference with *hisS *by spacer #1 almost certainly occurred during the recent evolutionary history of *P. carbinolicus*. It is likely that this interference resulted in a growth-limiting shortage of histidyl-tRNA. As expected under this selective pressure, genes with numerous and/or closely spaced histidine codons have evidently been counterselected in the genome of *P. carbinolicus*. Although loss or mutation of ancestral genes is easiest to surmise from comparative genomics, acquisition of low-histidine-demand genes and failure to acquire new high-histidine-demand genes also contribute to this difference.

Among the ancestral gene losses from the *P. carbinolicus *genome attributable to interference with *hisS *by spacer #1, the most obvious are the multiheme *c*-type cytochromes typical of *Geobacter *species. The *P. carbinolicus *genome encodes only 14 *c*-type cytochromes [36], whereas the *D. acetoxidans *genome encodes at least 80 such genes (M. A., unpublished). These cytochromes are not well conserved even between closely related *Geobacter *species [33,63], and it has been hypothesized that they have a generic function as capacitors [64]. Therefore, in addition to the loss of eight ancestral multiheme *c*-type cytochrome families by *P. carbinolicus*, the non-evolution of new families may also be an effect of interference with *hisS*.

Of the other eight families of ancestral genes that exhibit loss or reduced histidine demand in *P. carbinolicus*, two have especially interesting metabolic and physiological implications. The *nuoL-1 *gene encodes a subunit of NADH dehydrogenase I with a conserved histidine cluster, implicated in proton pumping. Abortive expression of *nuoL-1 *due to interference with *hisS *by spacer #1 in an ancestor of *P. carbinolicus*, leading to a loss of function for the entire NADH dehydrogenase I complex, would have favoured the elimination of all fourteen structural genes of the complex from the genome, which is what has occurred. The reason why this deletion was not lethal is probably that another ancestral NADH dehydrogenase I complex, for which the *nuoL-2 *gene does not contain a cluster of histidine codons, has been retained by *P. carbinolicus*. Conceivably, loss of a major respiratory enzyme complex by *P. carbinolicus *caused it to rely more on laterally acquired fermentative pathways.

Interference with *hisS *by spacer #1 also offers an explanation for the diminutive histidine cluster in the *znuA *gene product of *P. carbinolicus*, which functions to bind zinc in the periplasm for active transport into the cell. Zinc is a cofactor of key metabolic enzymes such as carbonic anhydrase, acetate kinase and phosphotransacetylase [65-67]. If the mutations in the histidine cluster of *znuA *reduce the efficiency of zinc uptake, it could have an impact on metabolism related to the unexplained inability of *P. carbinolicus *to oxidize acetate [28] despite the presence of a complete set of tricarboxylic acid cycle genes in the genome.

## Conclusions

This paper reports genetic and comparative genomic evidence that housekeeping genes can be targets of chronic CRISPR interference. Spacer #1 is shown to be transcribed into RNA with comparable amounts of both strands in *P. carbinolicus*, and to inhibit the growth of a transgenic strain of *G. sulfurreducens *that relies on *hisS *of *P. carbinolicus*, without drastically reducing the level of *hisS *RNA. The genome of *P. carbinolicus *exhibits the expected effect of a histidyl-tRNA shortage. Overall, the ancestral genes lost or mutated by the *P. carbinolicus *genome as part of its tendency towards fewer histidines per gene and lower histidine demand illustrate the de-emphasis of the metal-respiring metabolism that is typical of other *Geobacteraceae*. More generally, while previous studies have approached CRISPR as a microbial immune system, another important role of the system may be to exert pressure on endogenous and essential genes, resulting in dramatic changes in the genome content and physiology of the host species.

## Abbreviations

cDNA: complementary deoxyribonucleic acid; CRISPR: clustered regularly interspaced short palindromic repeats; DNA: deoxyribonucleic acid; IPTG: isopropylthio-*B*-D-galactopyranoside; PCR: polymerase chain reaction; QRT-PCR: quantitative real-time polymerase chain reaction; RNA: ribonucleic acid; tRNA: transfer ribonucleic acid.

## Authors' contributions

MA conceived of the study, performed experiments and wrote the manuscript. DL offered guidance. Both authors read and approved the final manuscript.

## Supplementary Material

Additional file 1 **Table S1**. Analysis of gene families of *P. carbinolicus*, *D. acetoxidans *and three *Geobacter *species for which one or more members have histidine content or histidine demand above the cutoffs (35 histidines or demand index 5.0). Click here for file

## References

[B1] Godde JS Bickerton A The repetitive DNA elements called CRISPRs and their associated genes: evidence of horizontal transfer among prokaryotes J Mol Evol 2006 62 6 718 729 10.1007/s00239-005-0223-z 16612537

[B2] Grissa I Vergnaud G Pourcel C The CRISPRdb database and tools to display CRISPRs and to generate dictionaries of spacers and repeats BMC Bioinformatics 2007 8 172 10.1186/1471-2105-8-172 17521438 PMC1892036

[B3] Haft DH Selengut J Mongodin EF Nelson KE A guild of 45 CRISPR-associated (Cas) protein families and multiple CRISPR/Cas subtypes exist in prokaryotic genomes PLoS Comput Biol 2005 1 6 e60 10.1371/journal.pcbi.0010060 16292354 PMC1282333

[B4] Jansen R Embden JD Gaastra W Schouls LM Identification of genes that are associated with DNA repeats in prokaryotes Mol Microbiol 2002 43 6 1565 1575 10.1046/j.1365-2958.2002.02839.x 11952905

[B5] Lillestøl RK Redder P Garrett RA Brugger K A putative viral defence mechanism in archaeal cells Archaea 2006 2 1 59 72 10.1155/2006/542818 16877322 PMC2685585

[B6] Makarova K Grishin N Shabalina S Wolf Y Koonin E A putative RNA-interference-based immune system in prokaryotes: computational analysis of the predicted enzymatic machinery, functional analogies with eukaryotic RNAi, and hypothetical mechanisms of action Biology Direct 2006 1 1 7 10.1186/1745-6150-1-7 16545108 PMC1462988

[B7] Makarova KS Aravind L Grishin NV Rogozin IB Koonin EV A DNA repair system specific for thermophilic Archaea and bacteria predicted by genomic context analysis Nucleic Acids Res 2002 30 2 482 496 10.1093/nar/30.2.482 11788711 PMC99818

[B8] Mojica FJ Diez-Villasenor C Garcia-Martinez J Soria E Intervening sequences of regularly spaced prokaryotic repeats derive from foreign genetic elements J Mol Evol 2005 60 2 174 182 10.1007/s00239-004-0046-3 15791728

[B9] Mojica FJ Diez-Villasenor C Soria E Juez G Biological significance of a family of regularly spaced repeats in the genomes of Archaea, Bacteria and mitochondria Mol Microbiol 2000 36 1 244 246 10.1046/j.1365-2958.2000.01838.x 10760181

[B10] Bolotin A Quinquis B Sorokin A Ehrlich SD Clustered regularly interspaced short palindrome repeats (CRISPRs) have spacers of extrachromosomal origin Microbiology 2005 151 Pt 8 2551 2561 10.1099/mic.0.28048-0 16079334

[B11] Pourcel C Salvignol G Vergnaud G CRISPR elements in *Yersinia pestis *acquire new repeats by preferential uptake of bacteriophage DNA, and provide additional tools for evolutionary studies Microbiology 2005 151 Pt 3 653 663 10.1099/mic.0.27437-0 15758212

[B12] Barrangou R Fremaux C Deveau H Richards M Boyaval P Moineau S Romero DA Horvath P CRISPR provides acquired resistance against viruses in prokaryotes Science 2007 315 5819 1709 1712 10.1126/science.1138140 17379808

[B13] Deveau H Barrangou R Garneau JE Labonte J Fremaux C Boyaval P Romero DA Horvath P Moineau S Phage response to CRISPR-encoded resistance in *Streptococcus thermophilus* J Bacteriol 2008 190 4 1390 1400 10.1128/JB.01412-07 18065545 PMC2238228

[B14] Brouns SJ Jore MM Lundgren M Westra ER Slijkhuis RJ Snijders AP Dickman MJ Makarova KS Koonin EV van der Oost J Small CRISPR RNAs guide antiviral defense in prokaryotes Science 2008 321 5891 960 964 10.1126/science.1159689 18703739 PMC5898235

[B15] Marraffini LA Sontheimer EJ CRISPR interference limits horizontal gene transfer in staphylococci by targeting DNA Science 2008 322 5909 1843 1845 10.1126/science.1165771 19095942 PMC2695655

[B16] Tang TH Bachellerie JP Rozhdestvensky T Bortolin ML Huber H Drungowski M Elge T Brosius J Huttenhofer A Identification of 86 candidates for small non-messenger RNAs from the archaeon *Archaeoglobus fulgidus* Proc Natl Acad Sci USA 2002 99 11 7536 7541 10.1073/pnas.112047299 12032318 PMC124276

[B17] Tang TH Polacek N Zywicki M Huber H Brugger K Garrett R Bachellerie JP Huttenhofer A Identification of novel non-coding RNAs as potential antisense regulators in the archaeon *Sulfolobus solfataricus* Mol Microbiol 2005 55 2 469 481 10.1111/j.1365-2958.2004.04428.x 15659164

[B18] Lillestøl RK Shah SA Brugger K Redder P Phan H Christiansen J Garrett RA CRISPR families of the crenarchaeal genus *Sulfolobus*: bidirectional transcription and dynamic properties Mol Microbiol 2009 72 1 259 272 10.1111/j.1365-2958.2009.06641.x 19239620

[B19] Hale C Kleppe K Terns RM Terns MP Prokaryotic silencing (psi)RNAs in *Pyrococcus furiosus* RNA 2008 14 12 2572 2579 10.1261/rna.1246808 18971321 PMC2590957

[B20] Semenova E Nagornykh M Pyatnitskiy M Artamonova II Severinov K Analysis of CRISPR system function in plant pathogen *Xanthomonas oryzae* FEMS Microbiol Lett 2009 296 1 110 116 10.1111/j.1574-6968.2009.01626.x 19459963

[B21] Carte J Wang R Li H Terns RM Terns MP Cas6 is an endoribonuclease that generates guide RNAs for invader defense in prokaryotes Genes Dev 2008 22 24 3489 3496 10.1101/gad.1742908 19141480 PMC2607076

[B22] Hale CR Zhao P Olson S Duff MO Graveley BR Wells L Terns RM Terns MP RNA-guided RNA cleavage by a CRISPR RNA-Cas protein complex Cell 2009 139 5 945 956 10.1016/j.cell.2009.07.040 19945378 PMC2951265

[B23] Viswanathan P Murphy K Julien B Garza AG Kroos L Regulation of *dev*, an operon that includes genes essential for *Myxococcus xanthus *development and CRISPR-associated genes and repeats J Bacteriol 2007 189 10 3738 3750 10.1128/JB.00187-07 17369305 PMC1913320

[B24] Zegans ME Wagner JC Cady KC Murphy DM Hammond JH O'Toole GA Interaction between bacteriophage DMS3 and host CRISPR region inhibits group behaviors of *Pseudomonas aeruginosa* J Bacteriol 2009 191 1 210 219 10.1128/JB.00797-08 18952788 PMC2612449

[B25] Sorek R Kunin V Hugenholtz P CRISPR--a widespread system that provides acquired resistance against phages in bacteria and archaea Nat Rev Microbiol 2008 6 3 181 186 10.1038/nrmicro1793 18157154

[B26] Lovley DR The microbe electric: conversion of organic matter to electricity Curr Opin Biotechnol 2008 19 6 564 571 10.1016/j.copbio.2008.10.005 19000760

[B27] Lovley DR Holmes DE Nevin KP Dissimilatory Fe(III) and Mn(IV) reduction Adv Microb Physiol 2004 49 219 286 10.1016/S0065-2911(04)49005-5 15518832

[B28] Lovley DR Phillips EJ Lonergan DJ Widman PK Fe(III) and S^0 ^reduction by *Pelobacter carbinolicus* Appl Environ Microbiol 1995 61 6 2132 2138 7793935 10.1128/aem.61.6.2132-2138.1995 PMC167486

[B29] Schink B Fermentation of 2,3-butanediol by *Pelobacter carbinolicus *sp. nov. and *Pelobacter propionicus *sp. nov., and evidence for propionate formation from C2 compounds Arch Microbiol 1984 137 33 41 10.1007/BF00425804

[B30] Haveman SA DiDonato RJ Jr Villanueva L Shelobolina ES Postier BL Xu B Liu A Lovley DR Genome-wide gene expression patterns and growth requirements suggest that *Pelobacter carbinolicus *reduces Fe(III) indirectly via sulfide production Appl Environ Microbiol 2008 74 14 4277 4284 10.1128/AEM.02901-07 18515480 PMC2493185

[B31] Richter H Lanthier M Nevin KP Lovley DR Lack of electricity production by *Pelobacter carbinolicus *indicates that the capacity for Fe(III) oxide reduction does not necessarily confer electron transfer ability to fuel cell anodes Appl Environ Microbiol 2007 73 16 5347 5353 10.1128/AEM.00804-07 17574993 PMC1950970

[B32] Methé BA Nelson KE Eisen JA Paulsen IT Nelson W Heidelberg JF Wu D Wu M Ward N Beanan MJ Genome of *Geobacter sulfurreducens*: metal reduction in subsurface environments Science 2003 302 5652 1967 1969 10.1126/science.1088727 14671304

[B33] Aklujkar M Krushkal J DiBartolo G Lapidus A Land ML Lovley DR The genome sequence of *Geobacter metallireducens*: features of metabolism, physiology and regulation common and dissimilar to *Geobacter sulfurreducens* BMC Microbiol 2009 9 109 10.1186/1471-2180-9-109 19473543 PMC2700814

[B34] Altschul SF Gish W Miller W Myers EW Lipman DJ Basic local alignment search tool J Mol Biol 1990 215 3 403 410 2231712 10.1016/S0022-2836(05)80360-2

[B35] Notredame C Higgins DG Heringa J T-Coffee: A novel method for fast and accurate multiple sequence alignment J Mol Biol 2000 302 1 205 217 10.1006/jmbi.2000.4042 10964570

[B36] Haveman SA Holmes DE Ding YH Ward JE Didonato RJ Jr Lovley DR *c*-type cytochromes in *Pelobacter carbinolicus* Appl Environ Microbiol 2006 72 11 6980 6985 10.1128/AEM.01128-06 16936056 PMC1636167

[B37] Holmes DE Nevin KP Lovley DR *In situ *expression of *nifD *in *Geobacteraceae *in subsurface sediments Appl Environ Microbiol 2004 70 12 7251 7259 10.1128/AEM.70.12.7251-7259.2004 15574924 PMC535187

[B38] Holmes DE Nevin KP O'Neil RA Ward JE Adams LA Woodard TL Vrionis HA Lovley DR Potential for quantifying expression of the *Geobacteraceae *citrate synthase gene to assess the activity of *Geobacteraceae *in the subsurface and on current-harvesting electrodes Appl Environ Microbiol 2005 71 11 6870 6877 10.1128/AEM.71.11.6870-6877.2005 16269721 PMC1287699

[B39] Coppi MV Leang C Sandler SJ Lovley DR Development of a genetic system for *Geobacter sulfurreducens* Appl Environ Microbiol 2001 67 7 3180 3187 10.1128/AEM.67.7.3180-3187.2001 11425739 PMC92998

[B40] Marx CJ Lidstrom ME Broad-host-range cre-lox system for antibiotic marker recycling in gram-negative bacteria Biotechniques 2002 33 5 1062 1067 12449384 10.2144/02335rr01

[B41] Butler JE Glaven RH Esteve-Nunez A Nunez C Shelobolina ES Bond DR Lovley DR Genetic characterization of a single bifunctional enzyme for fumarate reduction and succinate oxidation in *Geobacter sulfurreducens *and engineering of fumarate reduction in *Geobacter metallireducens* J Bacteriol 2006 188 2 450 455 10.1128/JB.188.2.450-455.2006 16385034 PMC1347312

[B42] Dehio M Knorre A Lanz C Dehio C Construction of versatile high-level expression vectors for *Bartonella henselae *and the use of green fluorescent protein as a new expression marker Gene 1998 215 2 223 229 10.1016/S0378-1119(98)00319-9 9714815

[B43] Marx CJ Lidstrom ME Development of improved versatile broad-host-range vectors for use in methylotrophs and other Gram-negative bacteria Microbiology 2001 147 Pt 8 2065 2075 11495985 10.1099/00221287-147-8-2065

[B44] Kunin V Sorek R Hugenholtz P Evolutionary conservation of sequence and secondary structures in CRISPR repeats Genome Biol 2007 8 4 R61 10.1186/gb-2007-8-4-r61 17442114 PMC1896005

[B45] Horvath P Romero DA Coute-Monvoisin AC Richards M Deveau H Moineau S Boyaval P Fremaux C Barrangou R Diversity, activity, and evolution of CRISPR loci in *Streptococcus thermophilus* J Bacteriol 2008 190 4 1401 1412 10.1128/JB.01415-07 18065539 PMC2238196

[B46] van der Ploeg JR Analysis of CRISPR in *Streptococcus mutans *suggests frequent occurrence of acquired immunity against infection by M102-like bacteriophages Microbiology 2009 155 Pt 6 1966 1976 10.1099/mic.0.027508-0 19383692

[B47] Mills S Griffin C Coffey A Meijer WC Hafkamp B Ross RP CRISPR analysis of bacteriophage-insensitive mutants (BIMs) of industrial *Streptococcus thermophilus *- implications for starter design J Appl Microbiol 2009 19709335 10.1111/j.1365-2672.2009.04486.x

[B48] Cui Y Li Y Gorge O Platonov ME Yan Y Guo Z Pourcel C Dentovskaya SV Balakhonov SV Wang X Insight into microevolution of *Yersinia pestis *by clustered regularly interspaced short palindromic repeats PLoS One 2008 3 7 e2652 10.1371/journal.pone.0002652 18612419 PMC2440536

[B49] Salzberg SL Sommer DD Schatz MC Phillippy AM Rabinowicz PD Tsuge S Furutani A Ochiai H Delcher AL Kelley D Genome sequence and rapid evolution of the rice pathogen *Xanthomonas oryzae *pv. *oryzae *PXO99A BMC Genomics 2008 9 204 10.1186/1471-2164-9-204 18452608 PMC2432079

[B50] Andersson AF Banfield JF Virus population dynamics and acquired virus resistance in natural microbial communities Science 2008 320 5879 1047 1050 10.1126/science.1157358 18497291

[B51] Tyson GW Banfield JF Rapidly evolving CRISPRs implicated in acquired resistance of microorganisms to viruses Environ Microbiol 2008 10 1 200 207 17894817 10.1111/j.1462-2920.2007.01444.x

[B52] Sissler M Delorme C Bond J Ehrlich SD Renault P Francklyn C An aminoacyl-tRNA synthetase paralog with a catalytic role in histidine biosynthesis Proc Natl Acad Sci USA 1999 96 16 8985 8990 10.1073/pnas.96.16.8985 10430882 PMC17719

[B53] Bond JP Francklyn C Proteobacterial histidine-biosynthetic pathways are paraphyletic J Mol Evol 2000 50 4 339 347 10795825 10.1007/s002399910037

[B54] Holt PJ Morgan DJ Sazanov LA The location of NuoL and NuoM subunits in the membrane domain of the *Escherichia coli *complex I: implications for the mechanism of proton pumping J Biol Chem 2003 278 44 43114 43120 10.1074/jbc.M308247200 12923180

[B55] Patzer SI Hantke K The ZnuABC high-affinity zinc uptake system and its regulator Zur in *Escherichia coli* Mol Microbiol 1998 28 6 1199 1210 10.1046/j.1365-2958.1998.00883.x 9680209

[B56] Methé BA Webster J Nevin K Butler J Lovley DR DNA microarray analysis of nitrogen fixation and Fe(III) reduction in *Geobacter sulfurreducens* Appl Environ Microbiol 2005 71 5 2530 2538 10.1128/AEM.71.5.2530-2538.2005 15870343 PMC1087574

[B57] Han D Krauss G Characterization of the endonuclease SSO2001 from *Sulfolobus solfataricus *P2 FEBS Lett 2009 583 4 771 776 10.1016/j.febslet.2009.01.024 19174159

[B58] Beloglazova N Brown G Zimmerman MD Proudfoot M Makarova KS Kudritska M Kochinyan S Wang S Chruszcz M Minor W A novel family of sequence-specific endoribonucleases associated with the clustered regularly interspaced short palindromic repeats J Biol Chem 2008 283 29 20361 20371 10.1074/jbc.M803225200 18482976 PMC2459268

[B59] Wiedenheft B Zhou K Jinek M Coyle SM Ma W Doudna JA Structural basis for DNase activity of a conserved protein implicated in CRISPR-mediated genome defense Structure 2009 17 6 904 912 10.1016/j.str.2009.03.019 19523907

[B60] Han D Lehmann K Krauss G SSO1450--a CAS1 protein from *Sulfolobus solfataricus *P2 with high affinity for RNA and DNA FEBS Lett 2009 583 12 1928 1932 10.1016/j.febslet.2009.04.047 19427858

[B61] Mojica FJ Diez-Villasenor C Garcia-Martinez J Almendros C Short motif sequences determine the targets of the prokaryotic CRISPR defence system Microbiology 2009 155 Pt 3 733 740 10.1099/mic.0.023960-0 19246744

[B62] Agari Y Sakamoto K Tamakoshi M Oshima T Kuramitsu S Shinkai A Transcription profile of *Thermus thermophilus *CRISPR systems after phage infection J Mol Biol 2010 395 2 270 281 10.1016/j.jmb.2009.10.057 19891975

[B63] Butler JE Young ND Lovley DR Evolution of electron transfer out of the cell: comparative genomics of six *Geobacter *genomes BMC Genomics 2010 11 40 10.1186/1471-2164-11-40 20078895 PMC2825233

[B64] Esteve-Nunez A Sosnik J Visconti P Lovley DR Fluorescent properties of *c*-type cytochromes reveal their potential role as an extracytoplasmic electron sink in *Geobacter sulfurreducens* Environ Microbiol 2008 10 2 497 505 10.1111/j.1462-2920.2007.01470.x 18093163

[B65] Katayama A Tsujii A Wada A Nishino T Ishihama A Systematic search for zinc-binding proteins in *Escherichia coli* Eur J Biochem 2002 269 9 2403 2413 10.1046/j.1432-1033.2002.02900.x 11985624

[B66] Matsuyama A Yamamoto-Otake H Hewitt J MacGillivray RT Nakano E Nucleotide sequence of the phosphotransacetylase gene of *Escherichia coli *strain K12 Biochim Biophys Acta 1994 1219 2 559 562 7918659 10.1016/0167-4781(94)90089-2

[B67] Smith KS Ferry JG Prokaryotic carbonic anhydrases FEMS Microbiol Rev 2000 24 4 335 366 10.1111/j.1574-6976.2000.tb00546.x 10978542

